# Systematic, continental scale temporal monitoring of marine pelagic microbiota by the Australian Marine Microbial Biodiversity Initiative

**DOI:** 10.1038/sdata.2018.130

**Published:** 2018-07-17

**Authors:** Mark V. Brown, Jodie van de Kamp, Martin Ostrowski, Justin R. Seymour, Tim Ingleton, Lauren F. Messer, Thomas Jeffries, Nahshon Siboni, Bonnie Laverock, Jaume Bibiloni-Isaksson, Tiffanie M. Nelson, Frank Coman, Claire H. Davies, Dion Frampton, Mark Rayner, Kirianne Goossen, Stan Robert, Bronwyn Holmes, Guy C.J. Abell, Pascal Craw, Tim Kahlke, Swan Li San Sow, Kirsty McAllister, Jonathan Windsor, Michele Skuza, Ryan Crossing, Nicole Patten, Paul Malthouse, Paul D. van Ruth, Ian Paulsen, Jed A. Fuhrman, Anthony Richardson, Jason Koval, Andrew Bissett, Anna Fitzgerald, Tim Moltmann, Levente Bodrossy

**Affiliations:** 1 School of Environmental and Life Sciences, University of Newcastle, Callaghan, NSW 2308, Australia.; 2 CSIRO Oceans and Atmosphere, Hobart, TAS 7004, Australia.; 3 Department of Chemistry and Biomolecular Sciences, Macquarie University, Sydney, NSW 2109, Australia.; 4 Plant Functional Biology and Climate Change Cluster, University of Technology, Sydney, Sydney, NSW 2007, Australia.; 5 Office of Environment and Heritage, Sydney, NSW 2000, Australia.; 6 Australian Centre for Ecogenomics, University of Queensland, Brisbane, QLD 4072, Australia.; 7 Hawkesbury Institute for the Environment, Western Sydney University, Sydney, NSW 2753, Australia.; 8 Geelong Centre for Emerging Infectious Diseases, Deakin University, Geelong, VIC 3220, Australia.; 9 CSIRO Oceans and Atmosphere, Brisbane, QLD 4067, Australia.; 10 Australian Institute of Marine Science, Darwin, NT 0810, Australia.; 11 Australian Institute of Marine Science, Townsville, QLD 4810, Australia.; 12 CSIRO Centre for Environment and Life Sciences, Floreat, WA 6014, Australia.; 13 South Australian Research and Development Institute, Adelaide, SA 5024, Australia.; 14 University of Southern California, Los Angeles, CA 90007, USA.; 15 Centre for Applications in Natural Resource Mathematics, School of Mathematics and Physics, The University of Queensland, St Lucia, QLD 4072, Australia.; 16 Ramaciotti Centre for Genomics, UNSW Australia, Sydney, NSW 2052, Australia.; 17 Bioplatforms Australia, Sydney, NSW 2109, Australia.; 18 Integrated Marine Observing System, Hobart, TAS 7004, Australia.

**Keywords:** Water microbiology, Marine biology, Sequencing

## Abstract

Sustained observations of microbial dynamics are rare, especially in southern hemisphere waters. The Australian Marine Microbial Biodiversity Initiative (AMMBI) provides methodologically standardized, continental scale, temporal phylogenetic amplicon sequencing data describing *Bacteria*, *Archaea* and microbial *Eukarya* assemblages. Sequence data is linked to extensive physical, biological and chemical oceanographic contextual information. Samples are collected monthly to seasonally from multiple depths at seven sites: Darwin Harbour (Northern Territory), Yongala (Queensland), North Stradbroke Island (Queensland), Port Hacking (New South Wales), Maria Island (Tasmania), Kangaroo Island (South Australia), Rottnest Island (Western Australia). These sites span ~30**°** of latitude and ~38**°** longitude, range from tropical to cold temperate zones, and are influenced by both local and globally significant oceanographic and climatic features. All sequence datasets are provided in both raw and processed fashion. Currently 952 samples are publically available for bacteria and archaea which include 88,951,761 bacterial (72,435 unique) and 70,463,079 archaeal (24,205 unique) 16 S rRNA v1-3 gene sequences, and 388 samples are available for eukaryotes which include 39,801,050 (78,463 unique) 18 S rRNA v4 gene sequences.

## Background & Summary

Bacteria, Archaea and microbial eukaryotes dominate ocean ecosystems, composing 90% of marine biomass^1^. They display a vast phylogenetic and functional diversity, and their combined metabolic activities control the biogeochemical cycles that drive critical ecosystem services. But our oceans are changing rapidly and are under threat from both natural and man-made stressors, including rising seawater temperatures, ocean acidification, over-exploitation of resources and increasing impacts from a growing tourism industry and higher population densities. Baseline census data concerning the identity and function of marine microbes provide a benchmark against which we can assess assemblage changes.

Australia has the 7th longest coastline in the world, stretching ~36 000 km. Over the last decade, sustained temporal observations of many oceanographic parameters around Australia’s coastline have been made possible by the government funded Integrated Marine Observing System (IMOS). IMOS was established in 2007, and operates a network of national reference stations (NRS) that consist of moored sensors and regular vessel-based sampling^2^ (for a description of the rationale, design and implementation of IMOS NRS see http://imos.org.au/fileadmin/user_upload/shared/ANMN/NRS_rationale_and_implementation_100811.pdf).

**Figure 1:**
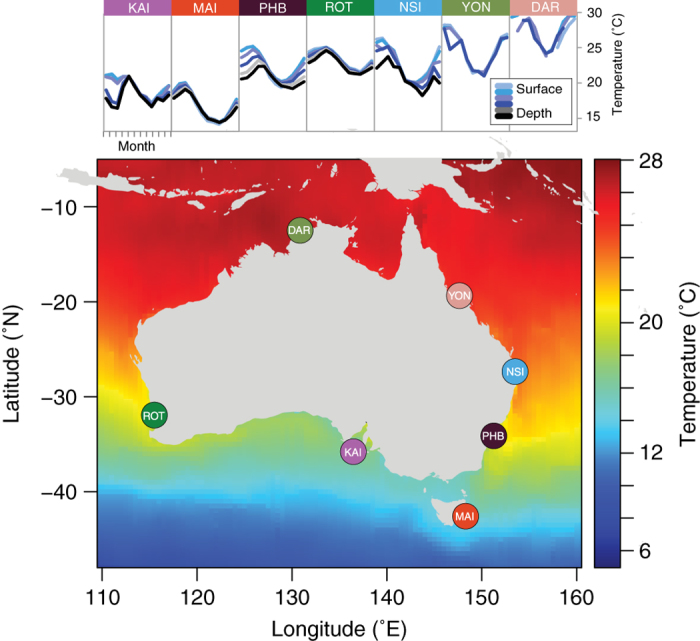
Locations of Australia’s Integrated Marine Observing System (IMOS) National Reference Stations (NRS). Inset are mean annual sea temperature plots for each month (1-12) and depth from which samples are collected at each station plotted over the range 10 **°**C to 32 **°**C, compiled with all CTD data collected during IMOS NRS cruises between June 2009-May 2016.

Marine microbial ecologists from a number of research institutions and universities have come together under the umbrella of the Australian Marine Microbial Biodiversity Initiative (AMMBI) to facilitate inclusion of microbial monitoring into the IMOS NRS infrastructure. Samples are collected at seven IMOS NRS: Port Hacking (site code: PHB), North Stradbroke Island (NSI), Maria Island (MAI) Rottnest Island (ROT), Yongala (YON), Darwin Harbour (DAR) and Kangaroo Island (KAI) (Fig. 1).

These IMOS NRS locations span over 30**°** of latitude and have been designed to maximize representation of the important marine biomes that are present around the continent^2^. Climatically they vary from tropical and monsoonal to cool temperate and highly seasonal habitats. All sites are affected to various degrees by basin scale events including the El Niño Southern Oscillation (ENSO) and the Indian Ocean Dipole (IOD), which are known to alter local oceanography, such as by enhancing upwelling^3^, and local biology, including fisheries^4^. Furthermore, these inter-annual events have a large influence on Australia’s two southerly flowing boundary currents, the Leeuwin Current (LC) on the Indian Ocean bounding west coast, and the East Australia Current (EAC) on the Pacific Ocean bounding east coast^5^. Both these currents transport warm, oligotrophic waters from the tropics to temperate latitudes. Changes in the extent and timing of southerly flow from these currents are having a large impact on both water temperatures^6^ and species ranges in southern Australia^7,8^, influencing fisheries^9^ and endangering native populations. However, little is known concerning the movements, adaption or range expansion of marine microbes.

## Methods

### Microbial sample collection

IMOS NRS sampling occurs using a variety of ocean going vessels dependent on local conditions. At all NRS, water samples are collected from between three and six pre-determined depths (depending on the water column depth) using Niskin bottles on either lines or a rosette. For each depth, a funnel and dark, sterile bottle are rinsed with 0.5 L of the retrieved seawater, and the bottle filled. In cases where filtration is conducted on shore (within 2 hours of collection) bottles are stored in a cooler with ice, out of direct sunlight. Microbial cells are collected by filtration of 2 L seawater through 0.2 μm pore Sterivex GP filter (Millipore, Massachusetts. Cat. # SVGPL10RC), using a Masterflex L/S Compact peristaltic pump fitted with an L/S 8 channel head (Cole Parmer). Pump tubing is rinsed with ~200 ml seawater from the appropriate depth prior to cell collection. Pumping continues for 1 min after sample has cleared the filter to dry. Both ends of the filter are capped and placed in individual snap-lock bags and kept on ice until being stored at −80 **°**C.

All AMMBI samples are collected in conjunction with IMOS NRS sampling (Fig. 1), so they are embedded within an extensive, quantitative, physical, chemical and biological oceanographic context. Contextual datasets from NRS are available for public search and download through the IMOS curated Australian Ocean Data Network Portal (https://portal.aodn.org.au/). A snapshot of IMOS data that corresponds to samples presented in this manuscript is available on Figshare (Data Citation 1). Parameters measured include conductivity, temperature, density, turbidity, fluorescence, PAR, Secchi depth, total alkalinity, total dissolved inorganic carbon, zooplankton dry weight, zooplankton size class, phytoplankton biomass, flow cytometry phytoplankton counts, phyto- and zooplankton microscopy identification and counts, HPLC pigment analysis, dissolved oxygen and nutrients including nitrite+nitrate, phosphate, silicate and ammonia. Further, *in-situ* moored sensor packages provide high-resolution data including acoustic doppler current profilers (ADCP), enabling the oceanographic context at the time of sampling to be accurately described. In addition, at Maria Island, Darwin, Yongala and North Stradbroke Island, surface expressions provide real-time meteorology, including wind-speed, direction, barometric pressure, humidity, air temperature and precipitation.

### Sampling sites

An overview of the locations, depths and bioregions sampled and data produced by this project is provided in Table 1. All sites are located on the Australian continental shelf.

**Darwin NRS** is located 4 nautical miles (nm) offshore, at a depth of 20 m (full tide) over sandy substrate in Darwin Harbour, a large inverse estuary near the city of Darwin, Northern Territory (population 116,215). It is in the tropical north with a monsoonal climate where the wet season occurs between December and April. This, the northernmost NRS in the network, occupies a broad, shallow, shelf-sea region, part of the Arafura-Timor Sea. During the Austral winter or “dry season”, oceanic waters predominate and the site is vertically well mixed. During the Austral summer or ‘wet season”, due to rainfall and terrestrial inputs, the water column can become highly stratified across periods from a few days up to a few weeks. The site has a large diurnal tidal movement of up to 8 m and significant amounts of suspended sediments. Samples are collected over a 12 h period during sampling days and mid tide samples used for microbial diversity and associated biogeochemical analyses.

Based on the Australian government bioregionalisation studies, Australia has six phytoplankton provinces^10^. Darwin NRS is located in phytoplankton province 1: diatom dominated shelf waters of the north-west Australia, the gulf of Carpentaria, Arafura Sea and Timor Seas.

**Yongala** NRS is located 11 nm offshore at a depth of 28 m over sandy substrate, mid-shelf between the mainland and the coral reef in the tropical Great Barrier Reef (GBR) Lagoon. The mooring is located near the Yongala Wreck, out from Cape Bowling Green, in the central GBR. The closest major city is Townsville (population 171 824), approximately 100 km to the north. The site is generally well mixed with competing influences of the south-eastward lagoonal branch of the EAC and the opposing south-easterly trade wind forced coastal current. However, the monsoonal climate brings rain during December to April, and may result in river plume impingement and stratification. This is a site where significant ocean acidification projects are being carried out.

Phytoplankton province 3: fast growing nanoplankton diatom dominated shallow waters of the Great Barrier Reef Lagoon.

**North Stradbroke Island NRS** is located 6.6 nm north east of North Stradbroke Island at a depth of 60 m over sandy substrate. It is 30 km southeast of the major city of Brisbane, Queensland (population 2.099 million), at the opening to large, shallow, Moreton Bay. The site is impacted by the southerly flowing EAC and its eddies, which may cause periodic nutrient enrichment through upwelling. This latitude is the biogeographic boundary for many tropical and subtropical species. The water column is well mixed between May-August and stratified for the remainder of the year and salinity may at times be affected by floodwaters from the nearby Brisbane River outflow.

Phytoplankton province 3: fast growing nanoplankton diatom dominated shallow waters of the Great Barrier Reef Lagoon

**Port Hacking NRS** is located 3 nm offshore at 100 m depth over fine muddy sand, near the major city of Sydney, New South Wales (population 4.3 million). This is a sub-tropical to temperate location with strong seasonality. The site is just downstream of the EAC separation zone and is impacted by the dynamics of flow of EAC and its eddy field. The water column is very well mixed between May and Sept (although the duration of this mixing has decreased in recent years (Ingleton, T. unpublished observation)) and highly stratified between December and March. Upwelling can occur via eddies or wind driven slope water intrusions. There is a long historical oceanographic dataset from this site (with intermittent gaps in certain parameters) dating back to 1953.

Phytoplankton province 4: the productive temperate neritic province comprising coastal waters of New South Wales, Tasmania, Victoria and South Australia.

**Maria Island NRS** is located 4 nm offshore from Maria Island at 80 m depth over muddy sand, on the eastern coast of Tasmania. This is the southernmost NRS. The nearest population centers are Orford (population 485) and Triabunna (population 796). The site is seasonally impacted by the most southerly extent of the EAC, which has been increasing over the past 60 years, resulting in rapid ocean warming in this region^6^. MAI may also be influenced seasonally by the LC. The water column is well mixed year-round with some slight stratification between November and March. There is a long historical oceanographic dataset from this site (with intermittent gaps in certain parameters) dating back to 1944.

Phytoplankton province 4: the productive temperate neritic province comprising coastal waters of New South Wales, Tasmania, Victoria and South Australia

**Kangaroo Island NRS** is located 4.5 nm west of Kangaroo Island, at 110 m depth over medium- to coarse-grained carbonate sand with conspicuous large bryozoan and gastropod skeletons, on a broad shelf in the Great Australian Bight, 112 km southwest of Adelaide (population 1.3 million). The NRS site is subject to episodic upwelling in the summer/autumn and is impacted by the eastward flow of the warm LC and outflow of dense Spencer Gulf water during the winter. Summer waters are stratified, with warm surface waters overlying a pool of cool enriched water upwelled from the Flinders Current (a northern boundary current) which flows westward along the continental slope. A significant volume of cooler water is forced above the critical depth, promoting high productivity. Winter waters are impacted by a significant outflow of dense Spencer Gulf water, so are well mixed, downwelling dominates, and productivity is significantly reduced.

Phytoplankton province 4: the productive temperate neritic province comprising coastal waters of New South Wales, Tasmania, Victoria and South Australia

**Rottnest Island NRS** is located 18 nm offshore and 2.5 nm from Rottnest Island, at a depth of 50 m over sand but surrounded by low relief limestone reef which is predominately covered with macro-algae, mainly Ecklonia spp, on the Western Australia coast, near Perth (population 1.83 million). This is the only NRS situated on the Indian Ocean coastline. This station is heavily impacted by the LC, which delivers warm but less saline waters southward. The LC is strongly affected by ENSO and IOD events. The prevailing winds and current direction leads to coastal suppression of upwelling along the Western Australian coast throughout most of the year, and consequently sea surface temperatures of up to 4–5 °C warmer than upwelling systems at similar latitudes elsewhere on the globe^2^. There is an historical oceanographic dataset from this site (with intermittent gaps in certain parameters) dating back to 1951.

Phytoplankton province 2: the tropical neritic communities carried southwards by the Leeuwin Current

### DNA extraction

All samples are couriered on dry ice, or in dewars filled with liquid nitrogen, to the Commonwealth Scientific and Industrial Research Organisation Oceans & Atmosphere (CSIRO O&A) laboratories in Hobart, Tasmania. DNA is extracted and purified using the PowerWater® Sterivex™ DNA Isolation Kit (MOBIO laboratories, Carlsbad, CA), following a slightly modified version of the manufacturer’s instructions^11^. The quality and quantity of DNA is checked using a NanoDrop™ 8000 Spectrophotometer (Thermo Scientific™) and DNA aliquot into multiple plates, vacuum dried and archived at -80 °C.

Further documentation outlining the standard operating procedures for sampling and DNA extraction is available at https://data.bioplatforms.com/organization/pages/bpa-marine-microbes/methods

### DNA sequence generation

Generation of sequence data for this project is supported by Bioplatforms Australia (BPA). All DNA amplification and sequencing is carried out at the Ramaciotti Centre for Genomics (UNSW Sydney, Australia).

Bacterial, archaeal and eukaryotic assemblages are surveyed using small-subunit ribosomal RNA gene amplicon sequencing methods. Amplicons are prepared using Bacterial 16 S rRNA gene primers 27 F – 519 R (refs 12,13), archaeal 16 S rRNA gene primers A2F/Arch21f – 519 R (refs 12,14) and eukaryotic 18 S rRNA gene primers TAReuk454FWD1 and TAReuk- Rev3 (ref. 15) (Table 2). (We note that the reverse primer used for the 18 S V4 region (TAReuk- Rev3) may discriminate against Haptophytes, a bias which should be considered when using the data, and which has been corrected in primer sets described by Piredda *et al.*^16^).

Amplicons are sequenced using the duel indexed paired end approach on the illumina MiSeq platform according to the manufactures instructions. Further documentation outlining the standard operating procedures for generating and sequencing amplicons is available at https://data.bioplatforms.com/organization/pages/bpa-marine-microbes/methods.

### DNA sequence processing

To allow for the highest possible phylogenetic resolution, data are provided as single nucleotide variants. Sequences are analyzed in a strictly standardized fashion alongside other Australian microbial biodiversity initiatives including the Biomes of Australian Soils (BASE) project^17^ enabling direct comparison and integration.

For each amplicon, each plate of illumina data is submitted to the following workflow separately, to identify correct biological sequences and build sample-by-read abundance matrices for samples on that plate^18^.

1. Illumina R1 and R2 reads are merged using FLASH^18,19^.
2. FASTA format sequences are extracted from FASTQ files and those<400 base pairs (bp) in length (or<350 bp for the 18 S amplicon) or containing N's or homopolymer runs of>8 bp are removed using MOTHUR, (v1.34.1)^20,21^.
3. Using the USEARCH 64 bit v10.0.240 (ref. 22) package, sequences are de-replicated (-derep_fulllength command), ordered by abundance and sequences with<4 representatives removed (-sortbysize command)
4. Chimeras are removed and biologically correct, zero-radius operational taxonomic units (zOTUs) identified (unoise3 command).
5. All quality-filtered sequences (from (2)) are then mapped to chimera-free zOTUs and a sample by read abundance table created using (-usearch_global)
6. Sample-by-read abundance matrices from each plate are merged based on the unique zOTU sequence, providing a sample-by-read abundance matrix for all samples in the dataset.
7. A final sample-by-read abundance data matrix is created by discarding sequences that are either not identified or not identified correctly for the given assay at the kingdom and phylum level (note this does not remove chloroplasts or mitochondrion from the Bacteria assay).
8. zOTUs are taxonomically classified with the SILVA v132 (ref. 23) database using MOTHUR’s implementation of the Wang classifier^24^ and a 60% bayesian probability cut-off. Eukaryotic zOTUs are also taxonomically classified using the Protist Ribosomal Reference database (PR2)^25^.
9. zOTUs can be readily clustered into OTUs of any similarity of the researchers choosing for comparison to other studies using a number of algorithms e.g. USEARCH cluster_fast^26^.

## Data Records

Each data record represents a molecular description of the relative abundance taxonomic profile of the microbial assemblage at a particular site, depth and time. Each record is associated with a sample and **e**ach sample is provided with a unique ID from the Australian National Data Service. All sequence data are made publically available through the National Center for Biotechnology Information (Data Citation 2). Currently datasets for 952 samples are publically available, which in total include 88,951,761 bacterial (representing 72,435 unique zOTUs) and 70,463,079 archaeal (24,205 zOTUs) small subunit rRNA gene sequences covering variable regions 1-3, and 39,801,050 (78,463 zOTUs) eukaryote small subunit rRNA gene sequences covering variable regions 4.

Paired end read (R1, R2) and indexed read (I1, I2) data in.fastq format are also available to download at https://data.bioplatforms.com/bpa/marine_microbes/amplicon.

All contextual datasets from IMOS NRSs are available for public search and download through the Australian Ocean Data Network Portal (https://portal.aodn.org.au/).

Files relating to Bacterial (labelled AMMBI_B16S_zotus_table_Silvav132_datarelease_20032018.txt), Archaeal (AMMBI_A16S_zotus_table_Silvav132_datarelease_20032018.txt) and Eukaryote (AMMBI_18 Sv4_zotus_table_Silvav132_PR2_datarelease_20032018.txt) datasets, which include zOTU sequence, sequentially indexed unique identifiers for each zOTU, sum of zOTU abundance in the dataset, zOTU sample by abundance table, SILVA v132 taxonomy string and PR2 taxonomy string (eukaryotes only) as well as a contextual data file (labelled AMMBI_Contextual.Data.Master.sheet20180131_NSD_submission) providing a snapshot of IMOS NRS environmental data harvested from the AODN at the time of manuscript submission are available on Figshare (Data Citation 1).

A search function enabling taxonomic and contextual data informed interrogation of, and download of data from, the latest sequence read abundance table is available from

https://data.bioplatforms.com/organization/about/australian-microbiome.

## Technical Validation

AMMBI represents, to our knowledge, the first methodologically standardized, continental-scale, temporal microbial ocean-observing network. We have attempted to minimize biases in the data generation pipeline by using the same simple sampling protocol at all stations and centralizing each step of DNA extraction (CSIRO), sequencing (Ramaciotti Centre for Genomics) and bioinformatics (CSIRO) in core facilities. That is, apart from sampling, all steps of the project are carried out concurrently on samples from all stations. The congruence of our phylogenetic characterization of samples with what is known concerning marine microbial assemblages globally reveals the suitability of our approach.

**Figure 2:**
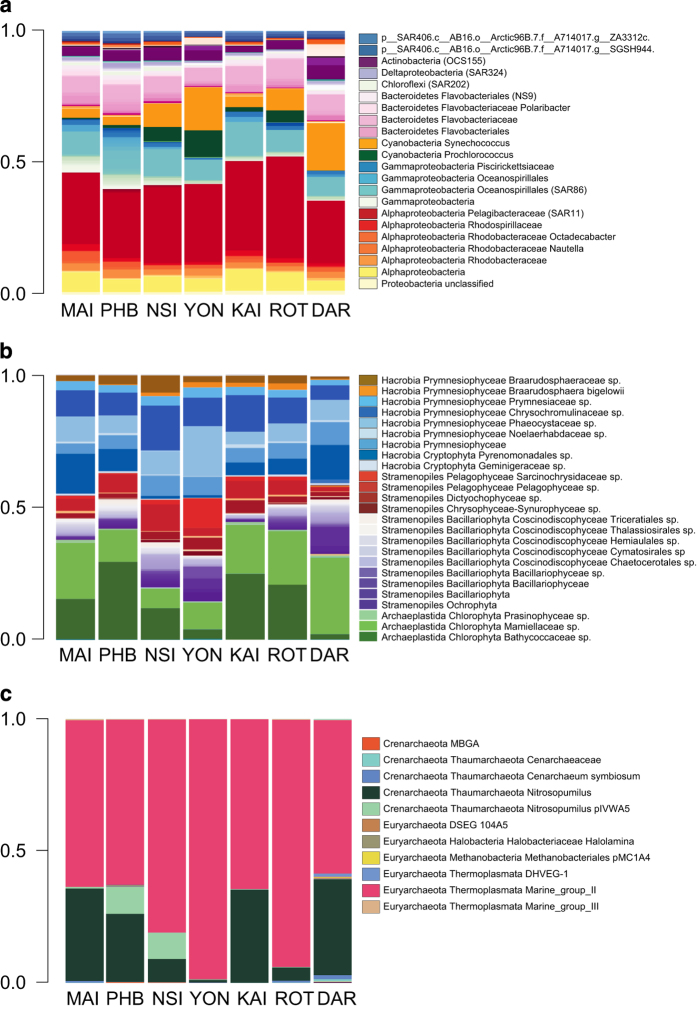
A molecular overview of microbial assemblages retrieved from surface waters at seven IMOS NRS around Australia. Profiles display the relative abundance of **a**) bacterial, **b**) plastid and **c**) archaeal taxa contributing to>0.1% of combined 16 S rRNA reads in samples collected at 0 m and 10 m depths over the course of the study. One two litre sample per depth was collected during each NRS sample trip (except Darwin where three samples per depth were taken at three hour intervals on two of the quarterly trips) and one DNA extraction and amplicon PCR performed per sample (MAI n= 81, PHB=87, NSI=90, YON=21, KAI=10, ROT=20, DAR=21). For this analysis, bacterial and archaeal taxonomic assignments were made using the GreenGenes database^30^ (release 13.5) and reads corresponding to chloroplast sequences were removed from the bacterial dataset and analysed independently with taxonomy assigned using the PhytoRef database based on 6,490 plastid 16 S rRNA gene sequences. Scripts used to generate Figs. 2 and 3 are available on Github at https://github.com/martinostrowski/marinemicrobes.

The alphaproteobacterial family the *Pelagibacteraceae* (SAR11 clade) compose ~30% of the sequences from surface waters at each station^27^, while the SAR86 clade is the most common and abundant gammaproteobacterial taxa (Fig. 2a). The marine cyanobacteria *Prochlorococcus* and *Synechococcus* constitute the bulk of the bacterial phototrophic assemblage and vary in their relative abundance, with *Prochlorococcus* relatively more prevalent at stations which display seasonal oligotrophic conditions, such as NSI and YON (Fig. 2a)^28^. Similarly, the Euryarchaeota Marine Groups II and Thaumarchaeota are the most abundant archaeal taxa^29^ (Fig. 2c).

**Figure 3:**
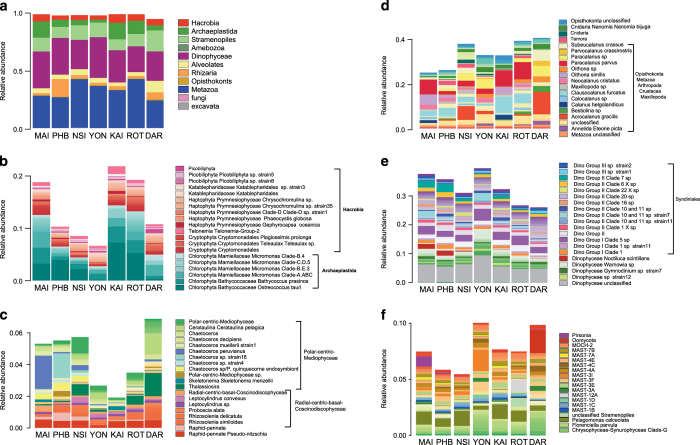
A molecular overview of eukaryotic taxa retrieved from surface waters at seven IMOS NRS around Australia. Profiles display the relative abundance of **a**) all taxa contributing to>0.1% of combined 18 S rRNA reads in samples collected at 0 m and 10 m depths over the course of the study. Sample details as per Fig. 2. More detailed taxonomic resolution is provided for **b**) the Archeaplastida and Hacrobia, (**c**) the Diatoms (Bacilliarophyta), (**d**) the Opisthokonts, (**e**) the Dinophyceae and (**f**) the Stramenopiles (other than diatoms). Note the scale differences for plots B-F which reflect the abundance of sequences for each subgroup in the entire dataset.

18S rRNA gene relative abundance data provides information concerning heterotrophic, symbiotic and parasitic eukaryotes including Metazoa and Amoebozoa, as well as mixotrophic and phototrophic taxa including Dinophyceae, Stramenopiles, Hacrobia (cryptomonads and haptophytes) and Archaeplastida (red and green algae) (Fig. 3). The overall diversity revealed within eukaryote taxa is exceptional (2,066 taxa identified). Analyses of taxa in sub-groups (Fig. 3b–f) reveals regional trends in species abundance and distribution. For example, several diatom taxa present distinct biogeographical preferences at individual NRS, particularly *Chaetoceros*, *Thalassiosira* and *Leptocylindrus*, while other species display a more cosmopolitan distribution, e.g. *Pseudo-nitzschia*. *Noctiluca scintillans,* a mixotrophic dinoflagellate linked to bright red and bioluminescent coastal blooms, is detected at stations along the East Coast (MAI, PHB and NSI; Fig. 3e). A number of species associated with harmful algal blooms are detected within the data (*Alexandrium, Noctiluca* and *Gymnodinium*) which highlights the potential of this initiative to enhance understanding of bloom dynamics within the context of a holistic record of microbial community structure.

Data on phototrophs is further enhanced by specific analysis of plastid 16 S rRNA genes that are retrieved using the bacterial 16 S gene assay (Fig. 2b). Plastid 16 S rRNA sequences comprise between 0.02 and 33.6% of total bacterial sequences in surface samples (<25 m depth). The plastid 16 S data is highly complementary with 18 S data and provides a proxy for the relative abundances of prokaryotic and eukaryotic phototrophs which can be used to fine-tune our understanding of regional phytoplankton provinces. For example, picoeukaryote species, *Ostreococcus* and *Micromonas* contribute a significant proportion of phytoplankton sequences, particularly at mid-latitude stations (MAI, KAI, ROT, PHB) while Cyanobacteria represent a greater proportion of 16 S sequences in low-latitude stations (DAR, YON, NSI).

## Usage Notes

There is considerable redundancy in the microbial taxa sampled across the seven NRS, enabling us to collect baseline data about the seasonal dynamics of a wide variety of organisms across the breadth of their habitat range. Hence the scope of this project enables us to effectively elucidate microbial niche conditions and describe microbial phenology. Further, the data provide a critical baseline against which to measure, and from which to predict, changes in microbial assemblages under future global change scenarios. Indeed, AMMBI data is being used to hindcast abundances of individual microbial taxa to long-term historic oceanographic datasets, to infer decadal scale trends in microbial composition, and to forecast taxa response to future climate scenarios. As observations are ongoing we will reveal any shifts in organismal dynamics and distributions which result from basin-scale climatic events such as the ENSO in the Pacific regions and the IOD which has its greatest affect along Australia’s western coast. We are also using this data to test the phylogenetic breadth of bioregionalisation provinces currently assigned using microscopy counts of phytoplankton.

We encourage users to download data from the Australian Microbiome searchable data portal at https://data.bioplatforms.com/organization/about/australian-microbiome. Full usage requires free registration. To search the marine dataset, navigate to “Tools/Search Facility”. Under the “Contextual Filters” box on the right use the “Environment” dropdown menu to choose “Marine”. Additional contextual filters (e.g. Temperature) can be “added” in this box, including using “NRS location code/ Voyage Code” to choose samples from specific NRS stations (e.g. text includes “MAI”). Users are also able to “Filter on Amplicon” (27f519r_bacteria, A2f519r_arcahea or 18Sv4F18Sv4R_v4_eukaryote) and/or taxonomy based on SILVAv132 6 string format.

DNA from all samples is archived in multiple aliquots at -80C at CSRIO Oceans and Atmosphere in Hobart, Tasmania and is accessible for re-analysis should the proposed methods be deemed to provide a substantial improvement or progress over prior results.

## Additional information

**How to cite this article**: Brown, M. V. *et al*. Continental scale monitoring of marine microbiota by the Australian Marine Microbial Biodiversity Initiative. *Sci. Data* 5:180130 doi: 10.1038/sdata.2018.130 (2018).

**Publisher’s note**: Springer Nature remains neutral with regard to jurisdictional claims in published maps and institutional affiliations.

## Supplementary Material



## Figures and Tables

**Table 1:** Details of sample set and data products from AMMBI.

Geographic Location (NRS Code)	Geoposition;	Depths Sampled (m)	Temporal regime; Range	Climatic Zone	Number of samples	BioProject
Darwin (DAR)	12**°** 24.00 S, 130**°** 46.08 E	0, 10, 20	Quarterly; August 2015 – May 2016	Tropical	30	PRJNA385736
Yongala (YON)	19**°** 18.51 S, 147**°** 37.10 E	0, 10, 20, 26	Monthly; June 2015 – May 2016	Tropical	41	PRJNA385736
North Stradbroke Island (NSI)	27**°** 20.50 S, 153**°** 33.73 E	0, 10, 20, 30, 40, 50	Monthly; June 2012 – May 2016	Subtropical	270	PRJNA385736
Port Hacking (PHB)	34**°** 05.00 S, 151**°** 15.00 E	0, 10, 25, 50, 75, 100	Monthly; June 2012 – May 2016	Subtropical	258	PRJNA385736
Maria Island (MAI)	42**°** 35.80 S, 148**°** 14.00 E	0, 10, 20, 40, 50, 75, 85	Monthly; July 2012 – May 2016	Temperate	263	PRJNA385736
Kangaroo Island (KAI)	35**°** 49.93 S, 136**°** 26.84 E	0, 10, 20, 50, 75, 100	Quarterly; June 2015 – May 2016	Subtropical	30	PRJNA385736
Rottnest Island (ROT)	32**°** 00.00 S, 115**°** 25.00 E	0, 10, 20, 30, 40, 46	Monthly; July 2015 – May 2016	Subtropical	60	PRJNA385736

**Table 2:** Locus specific primer sequences.

Primer Target: Name	Primer Sequences	Primer Reference
Bacteria 16S: 27f	AGAGTTTGATCMTGGCTCAG	^13^
Archaea 16S: A2f/Arch21f	TTCCGGTTGATCCYGCCGGA	^14^
Bacteria/Archaea 16S: 519r	GWATTACCGCGGCKGCTG	^12^
Eukaryote 18S: TAReuk454FWD1	CCAGCASCYGCGGTAATTCC	^15^
Eukaryote 18S: TAReuk- Rev3	ACTTTCGTTCTTGATYRATGATCTRYATC	^15^
